# Functional and structural similarity of human DNA primase [4Fe4S] cluster domain constructs

**DOI:** 10.1371/journal.pone.0209345

**Published:** 2018-12-18

**Authors:** Marilyn E. Holt, Lauren E. Salay, Elizabeth O’Brien, Jacqueline K. Barton, Walter J. Chazin

**Affiliations:** 1 Departments of Biochemistry and Chemistry, and Center for Structural Biology, Vanderbilt University, Nashville, Tennessee, United States of America; 2 Division of Chemistry and Chemical Engineering, California Institute of Technology, Pasadena, California, United States of America; Universität Stuttgart, GERMANY

## Abstract

The regulatory subunit of human DNA primase has a C-terminal domain (p58C) that contains a [4Fe4S] cluster and binds DNA. Previous electrochemical analysis of a p58C construct revealed that its affinity for DNA is sensitive to the redox state of the [4Fe4S] cluster. Concerns about the validity of this conclusion have been raised, based in part on differences in X-ray crystal structures of the p58C_272-464_ construct used for that study and that of a N-terminally shifted p58C_266-456_ construct and consequently, an assumption that p58C_272-464_ has abnormal physical and functional properties. To address this controversy, a new p58C_266-464_ construct containing all residues was crystallized under the conditions previously used for crystallizing p58C_272-464_, and the solution structures of both constructs were assessed using circular dichroism and NMR spectroscopy. In the new crystal structure, p58C_266-464_ exhibits the same elements of secondary structure near the DNA binding site as observed in the crystal structure of p58C_272-464_. Moreover, in solution, circular dichroism and ^15^N,^1^H-heteronuclear single quantum coherence (HSQC) NMR spectra show there are no significant differences in the distribution of secondary structures or in the tertiary structure or the two constructs. To validate that the two constructs have the same functional properties, binding of a primed DNA template was measured using a fluorescence-based DNA binding assay, and the affinities for this substrate were the same (3.4 ± 0.5 μM and 2.7 ± 0.3 μM, respectively). The electrochemical properties of p58C_266-464_ were also measured and this p58C construct was able to engage in redox switching on DNA with the same efficiency as p58C_272-464_. Together, these results show that although p58C can be stabilized in different conformations in the crystalline state, in solution there is effectively no difference in the structure and functional properties of p58C constructs of different lengths.

## Introduction

DNA synthesis at the replication fork begins with the formation of 8–12 nucleotide (nt) RNA primers on the single-stranded DNA template [1, 2]. In eukaryotes, primers are generated by the heterotetrameric DNA polymerase α-primase (pol-prim) complex, which possesses two enzymatic activities in two distinct active sites [3–5]. Primase, a DNA-dependent RNA polymerase, generates the initial hybrid RNA-DNA primed substrate, which is then handed off to DNA polymerase α (pol α) to extend the initial primer by approximately twenty DNA nts. The extended primed substrates are in turn handed off to the processive polymerases ε and δ, which synthesize the bulk of nascent DNA on the leading and lagging strands, respectively [6–8].

Human DNA primase is composed of catalytic (p48) and regulatory (p58) subunits. The regulatory subunit has a C-terminal domain (p58C) that is unique to higher eukaryotes and contains a [4Fe4S] cluster [9–11]. This domain regulates the catalytic efficiency of primase, a function attributed to the ability to bind nucleotides, DNA template, and primed substrate [7, 9, 10, 12–18]. We have recently proposed that [4Fe4S] redox control of DNA binding affinity may serve as a mechanism to drive handoff of the RNA primed template from the primase to the pol α subunits of human pol-prim [19]. Skepticism about some of the reported results have been expressed and debated [20–22], much of which was related to differences in crystal structures obtained from p58C constructs with different N-termini [23].

**Fig 1:**
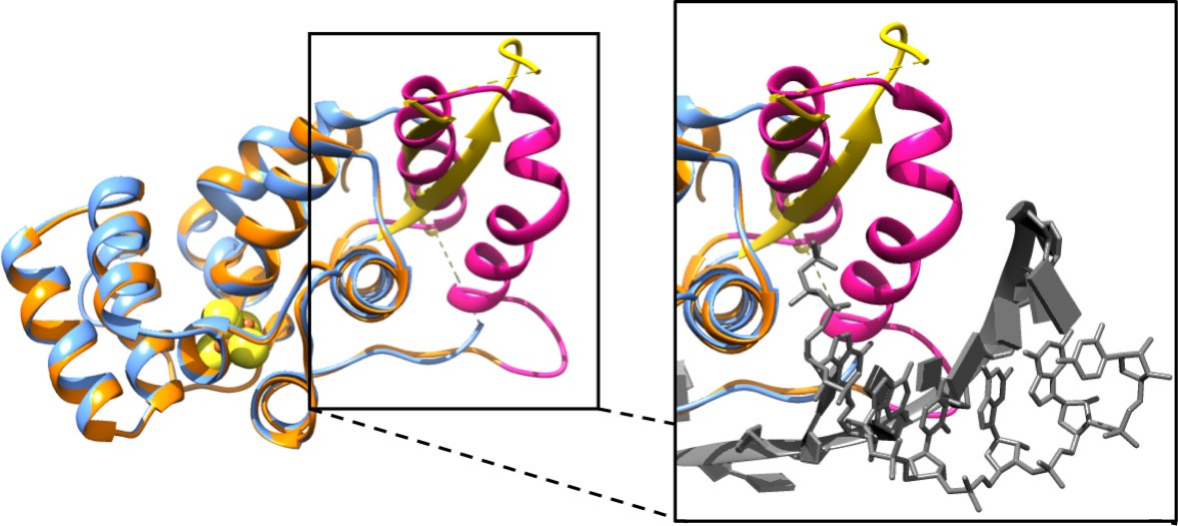
Comparison of the structures of p58C_272-464_ and p58C_266-456_. (A) Best-fit superposition over all backbone atoms of the crystal structures of the p58C_272-464_ (PDB ID: 3L9Q) and p58C_266-456_ (PDB ID: 5F0S) constructs. The substrate in the p58C_266-456_ structure is removed for clarity. Inset: Best-fit superposition over all backbone atoms of the DNA binding region with the primed substrate shown in the p58C_266-456_ structure. p58C_266-456_ is colored orange, p58C_272-464_ blue, and the RNA-primed DNA substrate in grey (RNA in sticks, DNA in slabs). In both panels, Leu318-His351 are colored yellow in the p58C_272-464_ structure and pink in the p58C_266-456_ structure. Connectivity between residues where electron density is missing is indicated by dashed lines.

The p58C domain of human primase has been crystallized under two different conditions and these have generated structures that have the same global fold but localized differences in secondary structures [14, 15]. Fig 1 shows a best-fit superposition of the high-resolution X-ray crystal structures of the two p58C_272-464_ and p58C_266-456_ constructs. The two structures are clearly very similar except for residues Leu318-His351, which are positioned near the DNA binding site. In the crystal structure of p58C_272-464_, these residues occupy a β-sheet-like structure that is stabilized by cross-strand interactions with another molecule in the unit cell [15]. In addition, a disulfide cross-link is formed during crystallization between the Cys449 residues of adjacent p58C molecules. In contrast, when p58C_266-456_ was crystallized under a different set of conditions, this β-type interaction is not observed and instead these residues occupy a helical hairpin [14]. It has been proposed that Ile271 is critical for stabilizing this helical motif and therefore the absence of this residue explains why p58C_266-456_ has a different structure [20, 22].

Many of the concerns about our work were attributed to the fact that the structural differences between the two p58C constructs occurred in a region that contains residues interacting with DNA substrates. However, it is well known that differences between crystal structures arise when crystals are formed under different crystallization conditions. In order to resolve any controversy and directly address the concerns raised about our findings, we report here a comprehensive set of studies of a p58C_266-464_ construct. Comparisons of the crystal structure of this construct are made to previously reported crystal structures. In addition, the structure was analyzed in solution by circular dichroism and NMR, along with assays of DNA binding affinities and electrochemical properties. These results show that the structure of Leu318-His351 varies in accord with crystallization conditions, whereas the structure in solution and biochemical properties of different p58C constructs are effectively the same.

## Methods

### p58C_266-464_ construct generation

The p58C_266-464_ construct was created with a Q5 site-directed mutagenesis kit from New England Biolabs, using the p58C_272-464_ construct plasmid [15] as the template, 5’- GGCAAGATTTCCTTAGATCAGATTGATTTGCTTTCTACC—3’ for the forward primer, and 5’–CACATTTCCgggcccctggaacagaac—3’ for the reverse primer. 10 ng of p58C_272-464_ plasmid was used in the exponential amplification, which was completed as described in the Q5 Site-Directed Mutagenesis Kit manual (New England Biolabs), except that the final extension time was extended to 5 minutes. The KLD reaction was also completed as described in the manual, except that 4 μL of PCR product was used in the reaction and the incubation time was increased to 20 minutes. 10 μL of the KLD reaction product was transformed into XL1-Blue cells. DNA was extracted from individual colonies with a Qiagen QIAprep Spin Miniprep Kit. Appropriate insertion of residues 266–271 was confirmed through plasmid sequencing (GENEWIZ, LLC).

### Protein expression and purification

p58C constructs were expressed and purified as previously described [19, 23]. In short, plasmid DNA was transformed into BL21 (DE3) cells (Novagen) and cultured in Terrific Broth media at 37°C to an OD_600_ of 0.6–0.8, when flasks were moved to a 21°C incubator with shaking. After 30 minutes, ferric citrate and ammonium ferrous citrate were added to a final concentration of 0.1 mg/mL, and isopropyl 1-thio-β-D-galactopyranoside was added to a final concentration of 0.5 mM. Protein was expressed at 21°C overnight. The same growth protocol was used to generate ^15^N-labeled p58C, except that cells were cultured in M9 media supplemented with ^15^N-labled ammonium chloride (Cambridge Isotopes) and expressed overnight at 25°C. The same purification scheme was used for both unlabeled and ^15^N-labeled protein [23]. In short, protein was first purified by nickel affinity chromatography (Amersham Biosciences). The 6xHis tag was cleaved with H3C protease and the protein was dialyzed into a low-imidazole buffer [19, 23]. The protein was repassed over the nickel column to remove the H3C protease and uncleaved protein. A heparin column was used as the final purification step to remove residual contaminants [19, 23].

### Crystallization and structure determination

The structure of p58C_266-464_ was determined as previously described for p58C_272-464_ [15, 23]. p58C_266-464_ crystals were grown by hanging drop vapor diffusion at 16°C from a drop composed of equal volumes of 50 mg/ml protein in 20 mM MES (pH 6.5) and 75 mM NaCl and reservoir solution containing 100 mM Tris (pH 8.5), 400 mM Li_2_SO_4_ and 18% (v/v) PEG 3350. Prior to data collection, crystals were transferred to a drop containing 100 mM Tris (pH 8.5), 400 mM Li_2_SO_4_,18% (v/v) PEG 3350, and 20% (v/v) glycerol for five seconds. The crystals were looped and flash frozen in liquid nitrogen. X-ray data were collected at beamline 21ID-D (Life Sciences Collaborative Access Team) of the Advanced Photon Source at Argonne National Laboratory at 11.5 kEV. All data were processed by HKL2000 [24]. The structure was determined using molecular replacement (PHASER-MR) with PDB entry 3L9Q, residues 274–316 and 360–457, as the search model. Manual model building for the structure was performed using *Coot* model building software, and waters were placed with the *Coot* routine, Find Waters [25]. The final model was obtained by iterative cycles of model building in *Coot* and structure refinement using Phenix.refine in the Phenix suite of programs [26, 27]. Structures were superimposed and RMSD calculated in Chimera with the Matchmaker algorithm [28]. Programs used for structure determination and refinement were accessed through SBGrid [29]. Statistics for data collection and refinement are shown in Table 1.

**Table 1:** Crystallographic data collection and refinement statistics.

***Data collection***	
Space Group	C2
Cell Dimensions	
a, b, c (Å)	110.19, 52.56, 88.77
α, β, γ (°)	90, 115.08, 90
Temperature (K)	100
Wavelength (Å)	1.08
Resolution (Å)	50.00–2.01 (2.08–2.01)
Unique Reflections	27193
R_meas_ (%)	0.14 (0.79)
*I/σI*	10.06 (2.12)
Completeness (%)	88.6 (84.4)
Redundancy	3.6 (3.3)
***Refinement***	
Resolution (Å)	50.00–2.01 (2.08–2.01)
No. reflections	27153
*R*_*work*_*/R*_*free*_	0.18/0.21 (0.23/0.24)
No. molecules/ASU	2
No. atoms	2872
Protein	2689
Ligand/ion	41
Water	52
*B-factors*	
Mean	39.1
Protein	38.7
Ligand/ion	66.9
R.m.s. deviations	
Bond lengths (Å)	0.003
Bond angles (°)	0.62
Ramachandran	
Favored	308 (99.4%)
Allowed	2 (0.6%)
Disallowed	0 (0%)

Values in parentheses are for the highest-resolution shell.

### Circular dichroism (CD) spectroscopy

Samples were buffer exchanged into 20 mM K_2_HPO_4_ (pH 7.2) and diluted to a concentration of 0.3 mg/mL. The far-UV CD spectrum over the range 190–260 nm was acquired at room temperature using a Jasco J-810 spectrophotometer. Each spectrum is the average of three scans acquired with a scanning rate of 50 nm/min and data pitch of 1 nm. Prior to generating the overlay in Graphpad Prism 7, the p58C_266-464_ spectrum was scaled to the p58C_272-464_ spectrum by averaging the values of the CD_208_(p58C_272-464_)/CD_208_(p58C_266-464_) and CD_222_(p58C_272-464_)/CD_222_(p58C_266-464_) ratios to generate a scaling factor (~0.86), then multiplying the entire p58C_266-464_ spectrum by this scaling factor.

### NMR spectroscopy

Spectra were acquired using a Bruker AV-III 800 MHz spectrometer equipped with a CPTCI single-gradient cryoprobe. ^15^N-enriched p58C constructs at a concentration of 200 μM were prepared in a buffer containing 20 mM MES (pH 6.5), 50 mM NaCl, 2 mM DTT, and 5% (v/v) D_2_O. Two-dimensional ^15^N-^1^H heteronuclear single quantum coherence (HSQC) spectra were acquired at 25°C with 2,048 and 128 points in the ^1^H and ^15^N dimensions, respectively. 64 scans were recorded in the direct (^1^H) dimension for each point sampled in the indirect (^15^N) dimension. Data were processed by Topspin (Bruker) and analyzed with Sparky (University of California).

### RNA primer generation

Triphosphorylated RNA primer was transcribed with T7 RNA polymerase [30] and purified on a 25% TBE-polyacrylamide gel supplemented with 8 M urea according to standard methods. Dried RNA pellet was resuspended in RNAse-free H_2_O and aliquoted prior to further purification. RNAs used for binding assays were HPLC purified on a Luna 5 μM C18(2) 100 Å 250X4.6 mm column (Phenomenex). Buffer A: 0.1 M ammonium formate; Buffer B: acetonitrile; flow rate: 1.5 mL/min. Purification program: 1–5% Buffer B over three minutes, 5–8% Buffer B over 22 minutes, then 80% Buffer B for five minutes. RNA typically eluted around 11 minutes. HPLC-purified RNA was further validated with mass spectrometry, which confirmed that a product of the expected mass had been generated.

### Substrate binding assays

The fluorescence intensity (FI) assay was performed with a Monolith NT.115 series microscale thermophoresis (MST) instrument (NanoTemper) at 25°C. Cy5-labeled 18 nt DNA template was purchased from Sigma-Aldrich. A 1.1:1 ratio of 8 nt RNA and this DNA template was annealed in annealing buffer (20 mM MES (pH 7.0), 75 mM NaCl), resulting in a 25 μM stock of annealed, primed substrate. This stock was diluted to 100 nM with MST buffer (20 mM MES (pH 6.5), 50 mM NaCl, 2 mM DTT, 0.05% Tween). The primed substrate was mixed with p58C and allowed to incubate in the dark at room temperature for 15 min. Samples with a final substrate concentration of 50 nM were then loaded into MO-K003 Monolith NT.115 hydrophobic capillaries (NanoTemper) and fluorescence was measured at 20% LED and 40% MST power. Final K_D_ values were calculated using the one-site total binding equation in GraphPad Prism 7. Titrations were completed after running an SD test to ensure that the concentration-dependent changes in fluorescence intensity were not due to adsorption to the capillaries or aggregation of the fluorophore [31].

MST RNA primer: 5’-PPP-GGCUCGGA-3’

MST DNA template: 5’-Cy5-AAACTCCGAGCCAACATA-3’

Fluorescence anisotropy (FA) was measured with a SpectraMax M5 microplate reader (Molecular Devices). A 6FAM-labeled 22 nt DNA template was purchased from Sigma-Aldrich. A 1.1:1 ratio of 12 nt RNA and this DNA template was annealed in annealing buffer (20 mM MES (pH 7.0), 75 mM NaCl), resulting in a 25 μM stock of annealed, primed substrate. The stock was diluted to 800 nM with DNA binding buffer (20 mM MES (pH 6.5), 50 mM NaCl, 2 mM DTT). This primed substrate was mixed with p58C and allowed to incubate in the dark at room temperature for 15 min. Samples with a final substrate concentration of 50 nM were then loaded into a 384-well plate and polarized fluorescence intensities were measured using excitation and emission wavelengths of 485 nm and 520 nm. The fluorescein control experiments were performed with 25 nM fluorescein (Sigma Aldrich) dissolved in DNA binding buffer containing 0.016% DMSO, which was then mixed with p58C and incubated in the dark at room temperature for 15 min prior to determining fluorescence anisotropy in the same way as for the p58C-DNA titrations. Three replicates were collected for each titration. Final K_D_ values were calculated using the one-site specific binding equation in GraphPad Prism 7; prior to using this equation, each binding curve was normalized by subtracting the fluorescence anisotropy value of the zero point from each point on the curve. K_D_ values are reported as the mean ± standard deviation of three measurements for each variant.

FA RNA primer: 5’-PPP-GGACCTCCAGGA-3’

FA DNA template: 5’-6FAM-AAACTCCTGGAGGTCCAACATA-3’

### Cyclic voltammetry (CV)

#### Sample preparation for electrochemistry

Multiplexed chips were fabricated as described previously [19]. p58C construct samples were stored prior to experiments in p58C storage buffer (20 mM Tris (pH 7.2), 75 mM NaCl). All p58C constructs were buffer exchanged into HEPES electrochemistry buffer (20 mM HEPES (pH 7.2), 75 mM NaCl) using Amicon ultra centrifugal filters (0.5 mL, 3 kDa MWCO) (Millipore Sigma). Protein was applied to the filter in volumes of 90–140 μL and centrifuged for 15 minutes at 14000 x g at 4°C. After centrifugation, 400 μL of HEPES electrochemistry buffer was applied to the filter and centrifuged at 14000 x g for 20 minutes. This was repeated four times to exchange p58C samples into HEPES electrochemistry buffer. After buffer exchange and recovery of sample by centrifugation (2 minutes, 1000 x g), concentrations of [4Fe4S] cluster-containing p58C or mutants were measured by using UV-Visible spectroscopy to monitor the absorbance of the [4Fe4S] cluster at 410 nm (extinction coefficient = 17000 M^-1^ cm^-1^) [19, 32]. Recovered samples (approx. 100–150 μL in volume) were deoxygenated for 2–3 minutes with argon. Samples were then transferred into the anaerobic chamber (Coy Laboratory products). Prior to deposition onto the gold electrode surface, p58C_266-464_ samples were diluted with previously deoxygenated HEPES electrochemistry buffer to a molar concentration of 40μM [4Fe4S] p58C. Samples were initially deposited onto multiplex chip quadrants in 20 μL volumes and the remaining sample deposited in a well of bulk solution above the chip surface, to a final volume of 200–300 μL.

#### p58C construct electrochemistry

All electrochemistry was performed using a CHI620D potentiostat and 16-channel multiplexer (CH Instruments) in an anaerobic glove chamber. Multiplex gold electrodes were part of a three electrode system with an external Ag/AgCl reference electrode (Bioanalytical Systems) and platinum counter electrode. Cyclic voltammetry scans were performed at a scan rate of 100 mV/s over a potential range of +0.412 V to -0.288 V vs. NHE or +0.512 V to -0.188 V vs NHE. Bulk electrolysis on DNA was performed at an applied potential of +0.512 V vs. NHE for all electrochemical oxidation reactions. The oxidizing potential was applied for at least 8.33 minutes for single oxidation reactions on a surface, and 6.67 minutes for the iterative oxidation cycles of p58C variants. The reducing potential was applied for 8.33 minutes in all electrochemical reduction reactions. All bulk electrolysis and cyclic voltammetry was performed in previously deoxygenated p58C electrochemistry buffer (20 mM HEPES (pH 7.2), 75 mM NaCl). Charge transfer (nC) in the cathodic peak of oxidized samples CV scans was assessed using the area under the current wave of the reduction signal. Charge transfer was measured for oxidized samples using CHI software, assessing the area under the reductive peak in CV after electrochemical oxidation. NTP-dependence of electrochemical signals was measured by pipetting a small volume (1–3 μL) of a 0.1 M ATP stock solution into each quadrant of the multiplexed chip setup. Samples were added by quadrant, as physical barriers in the setup prevent diffusion of NTPs between electrode quadrants. After the volume of ATP stock was deposited onto the electrode quadrant, resulting in a 2.5 mM or 5 mM concentration of ATP in the quadrant, CV scans were measured (100 mV/s scan rate). Charge transfer was assessed using CHI software; charge values were determined by calculation of the area under the reductive and oxidative peak curves. Midpoint potentials of NTP-dependent redox signals were assessed using the peak selection function in CHI software.

## Results

### p58C can be crystallized in different conformations

**Fig 2:**
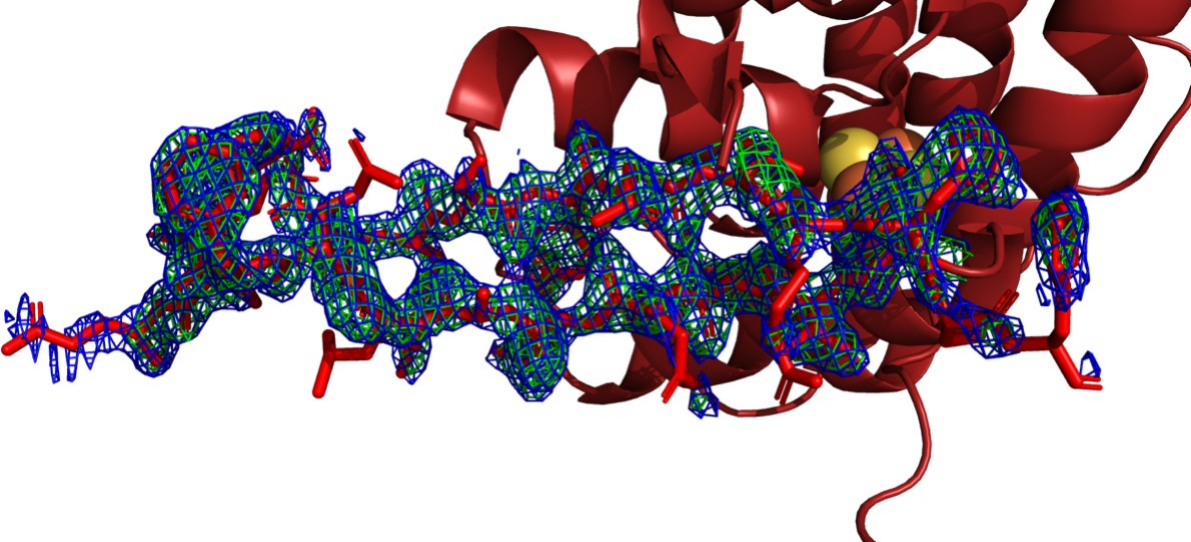
Electron density maps revealing β-sheet-like conformation for residues Leu318-His351. The ribbon diagram of the structure with Leu318-His351 displayed in stick representation is docked into the starting electron density map around the β-sheet-like region. Blue mesh represents the 2F_o_-F_c_ map contoured at 1 σ, Green mesh represents the F_o_-F_c_ map, contoured at 3 σ. Figure made in Pymol using the isomesh command [33].

To test if the differences between the p58C_266-456_ and p58C_272-464_ structures were a by-product of the differences in sequence, we produced, purified, and crystallized a p58C construct containing residues 266–464 in the conditions used to crystallize p58C_272-464_ [15, 19]. These crystals diffracted to 1.6 Å and the data were phased using molecular replacement with the structure of p58C_272-464_ (3L9Q). To avoid phase bias, residues 315–360 were excluded when defining the search model. With these residues omitted, density for residues in extended conformation that together formed a beta-type interaction were clearly evident in the p58C_266-464_ 2Fo-Fc map (Fig 2).

**Fig 3:**
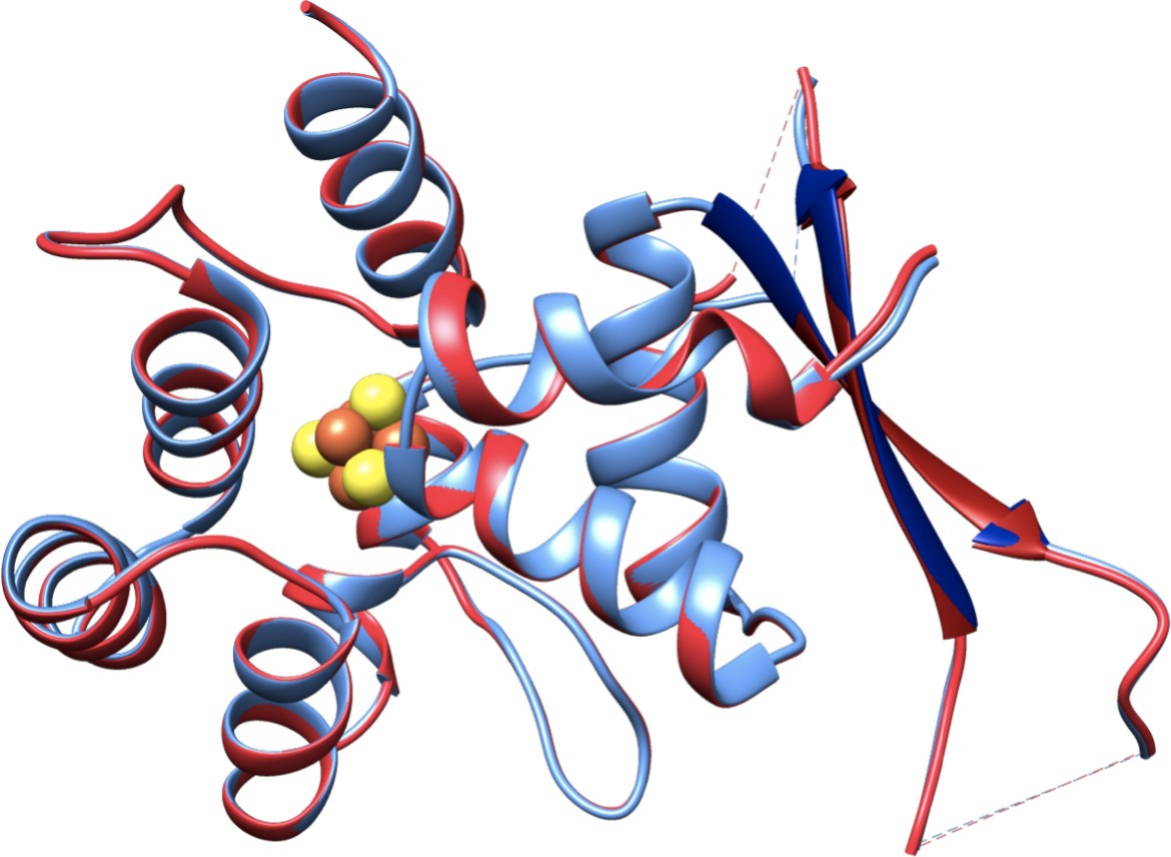
Comparison of the X-ray crystal structures of p58C_266-464_ and p58C_272-464_ obtained from crystals grown under identical conditions. Ribbon diagrams of the best-fit superposition over all backbone atoms of p58C_272-464_ (blue) and p58C_266-464_ (red) crystal structures. Residues Leu318-His351 are colored dark blue in the p58C_272-464_ structure and dark red in the p58C_266-464_ structure. Connectivity between residues where electron density is missing is indicated by dashed lines. The [4Fe4S] clusters are shown as the cluster of yellow and orange spheres.

This region was re-built manually in *Coot*, and the final structure was refined to 2.01 Å resolution. As previously observed for p58C_272-464_ under these conditions, crystallized as a dimer, with a disulfide cross-link between the two Cys449 residues and several stabilizing interactions between symmetry-related molecules (S2 Fig). In p58C_266-464_, residues 330–340 and 353–360 in chain A and 330–345 and 353–359 in chain B are missing due to disorder. Disordered residues in the same regions are observed in the structure of p58C_272-464_. A best-fit superposition of the two structures reveals they are very similar (Fig 3), with a backbone RMSD of only 0.23 Å. This finding shows that the differences in residues Leu318-His351 evident from comparing the previous p58C structures [14, 15, 18] are not intrinsic to the differences in the N-termini of the constructs but rather to differences in the crystallization conditions.

### CD and NMR show p58C_272-464_ and p58C_266-464_ are structurally similar in solution

**Fig 4:**
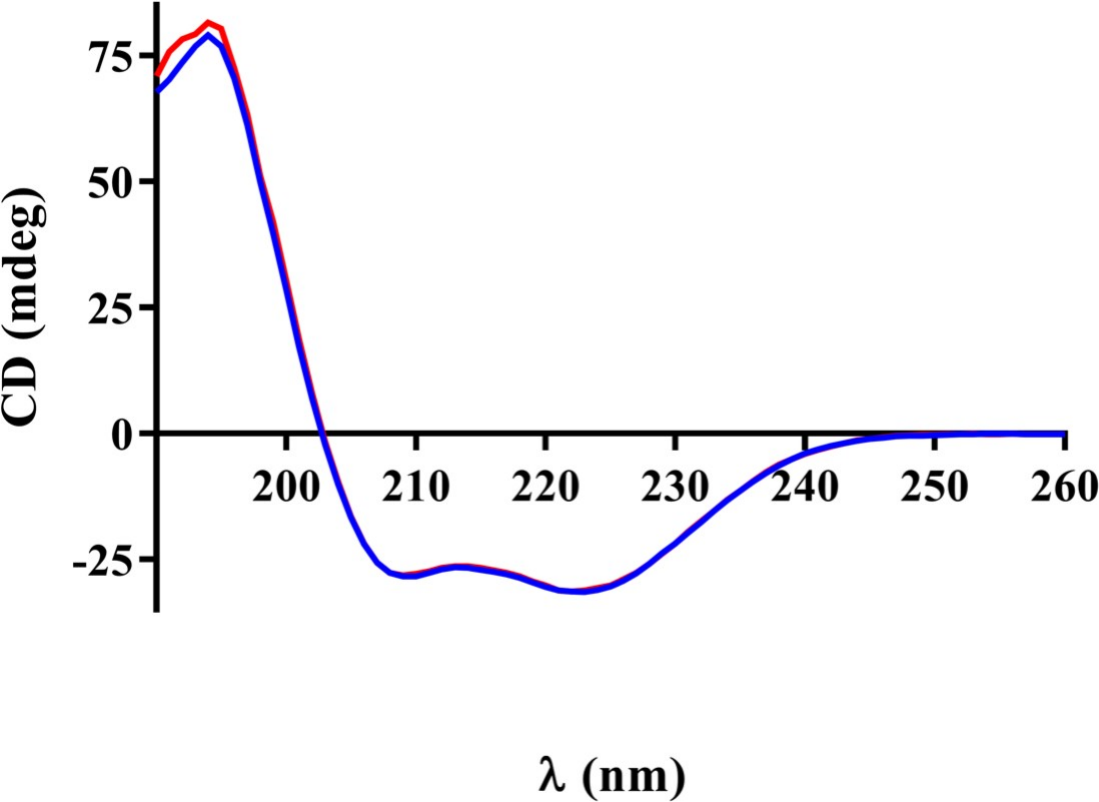
The p58C_272-464_ and p58C_266-464_ constructs have the same distribution of secondary structures in solution. CD spectra p58C_272-464_ (blue) and p58C_266-464_ (red) were collected at room temperature for a solution containing 0.3 mg/mL of protein in a buffer containing 20 mM K_2_HPO_4_ (pH 7.0).

**Fig 5:**
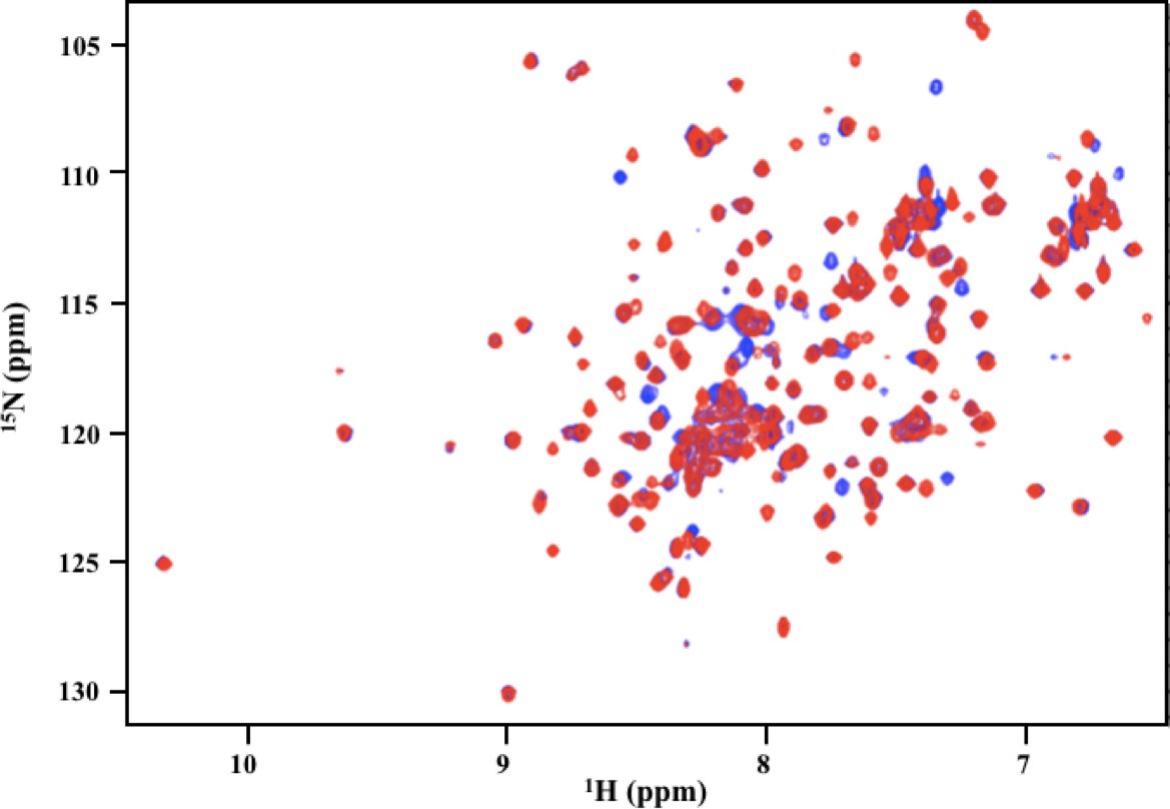
The tertiary structures of p58C_272-464_ and p58C_266-464_ constructs are similar in solution. ^15^N-^1^H HSQC NMR spectra of p58C_272-464_ (blue) and p58C_266-464_ (red) were acquired at 25°C on a Bruker AV-III spectrometer operating at 800 MHz. The samples contained 200 μM protein in a buffer containing 20 mM MES (pH 6.5), 50 mM NaCl, 2 mM DTT, and 5% ^2^H_2_O.

Because the differences in the X-ray crystal structures can be attributed to differences in the crystallization conditions, we used CD and NMR spectroscopy to determine if there are significant structural differences between p58C_272-464_ and p58C_266-464_ in solution. The CD spectra of p58C_272-464_ and p58C_266-464_ acquired under identical conditions were very similar (Fig 4), showing that the two constructs contain the same distribution of secondary structure [34]. To further investigate the structures of these constructs, ^15^N-^1^H HSQC spectra were collected for ^15^N-enriched p58C_272-464_ and p58C_266-464_. Fig 5 shows an overlay of HSQC spectra acquired under identical conditions for p58C_272-464_ and p58C_266-464_. These match well with previously published spectra [9, 15], indicating that both constructs are well-folded. There are a number of chemical shift perturbations between the two spectra, consistent with the presence of six additional N-terminal residues. Overall, the distribution of peaks is very similar in the two spectra, indicating that the overall tertiary structure of p58C_272-464_ and p58C_266-464_ are the same. Sequence-specific assignments could provide insight into the nature of the differences in the NMR spectra. However, the presence of the paramagnetic [4Fe4S] cluster causes extreme line broadening due to rapid relaxation of spatially proximate residues, greatly complicating efforts to obtain resonance assignments [35]. Despite the absence of assignments, because the NMR spectra are so similar, and the CD spectra are virtually identical, we can conclude that there are no substantial differences in the structures of p58C_272-464_ and p58C_266-464_ in solution.

### The p58C_272-464_ and p58C_266-464_ constructs have the same affinity for a primed template

**Fig 6:**
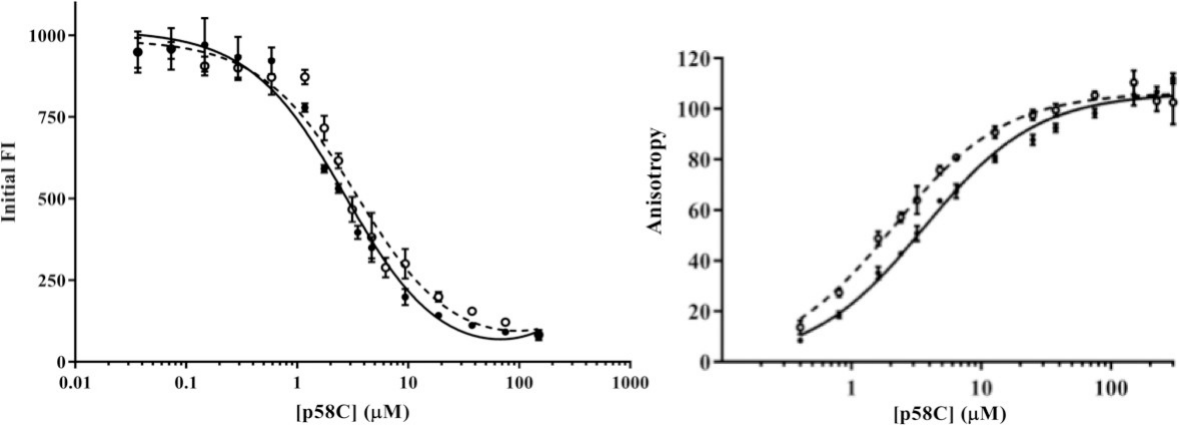
The two p58C constructs bind a RNA-primed substrate with the same affinity. Left: The p58C_272-464_ (filled circle, solid line) and p58C_266-464_ (open circle, dashed line) proteins were titrated into Cy5-labeled substrate in a buffer containing 20 mM MES (pH 6.5), 50 mM NaCl, 2 mM DTT, and 0.05% Tween. FI was measured at 25°C. RNA: 5’-GGCUCGGA-3’; DNA: 5’-Cy5-AAACTCCGAGCCAACATA-3’. Right: The p58C_272-464_ (filled circle, solid line) and p58C_266-464_ (open circle, dashed line) proteins were titrated into 6FAM-labeled substrate in a buffer containing 20 mM MES (pH 6.5), 50 mM NaCl, and 2 mM DTT. Fluorescence anisotropy was measured at 25°C. RNA: 5’-GGACCTCCAGGA-3’; DNA: 5’-6FAM-AAACTCCTGGAGGTCCAACATA-3’. For both panels, error bars represent the standard deviation of three replicates.

Having established that the structures of p58C_272-464_ and p58C_266-464_ are essentially the same, it is important to determine if the two constructs are functionally equivalent. Since DNA binding is an essential property for p58C function, we turned to fluorescence assays to compare the DNA binding properties of the two constructs. We first used a fluorescence intensity (FI)-based assay with an RNA-primed DNA substrate composed of a Cy5-labelled 18 nt DNA template strand annealed with a complementary 5’-triphosphorylated 8 nt RNA primer, corresponding to a high affinity product produced by primase [36]. The affinities of p58C_272-464_ and p58C_266-464_ for this substrate were measured by monitoring initial fluorescence intensity (FI) changes as p58C was titrated into a fluorescently-labelled substrate (Fig 6). Fitting these data to a single site binding equation returned dissociation constants (K_D_) within statistical error: 2.7 ± 0.3 μM and 3.4 ± 0.5 μM for p58C_272-464_ and p58C_266-464_, respectively.

Because the fluorescence intensity-based assay is measuring fluorescence quenching, it is conceivable that this assay could be sensing direct interaction with the probe. We turned to a fluorescence anisotropy (FA) assay to measure the binding affinity by an alternate approach and rule out the possibility of direct probe binding. For this assay, an RNA-primed DNA substrate was used composed of a 6FAM-labelled 22 nt DNA template strand annealed with a complementary 5’-triphosphorylated 12 nt RNA primer. We used the longer template to place the probe further away from the RNA-DNA hybrid region and minimize probe quenching. The K_D_ values of 3.6 ± 0.1 μM and 2.1 ± 0.1 μM obtained by FA for p58C_272-464_ and p58C_266-464_, respectively are very similar to those obtained by the fluorescence intensity assay (Fig 6). To test for direct binding of fluorescein to the p58C constructs, the FA assay was repeated with free fluorescein (see S6 Fig). The absence of binding of fluorescein in these control experiments confirms that the responses in the FI and FA assays are due to interactions between p58C and the DNA substrate. The narrow range (2.1–3.7 μM) and average values (3.2 μM and 2.8 μM, respectively) of K_D_ for the two constructs measured by the two approaches demonstrate there is no functionally significant difference in the DNA binding properties of these two constructs.

### The p58C_272-464_ and p58C_266-464_ constructs have similar electrochemical properties

**Fig 7:**
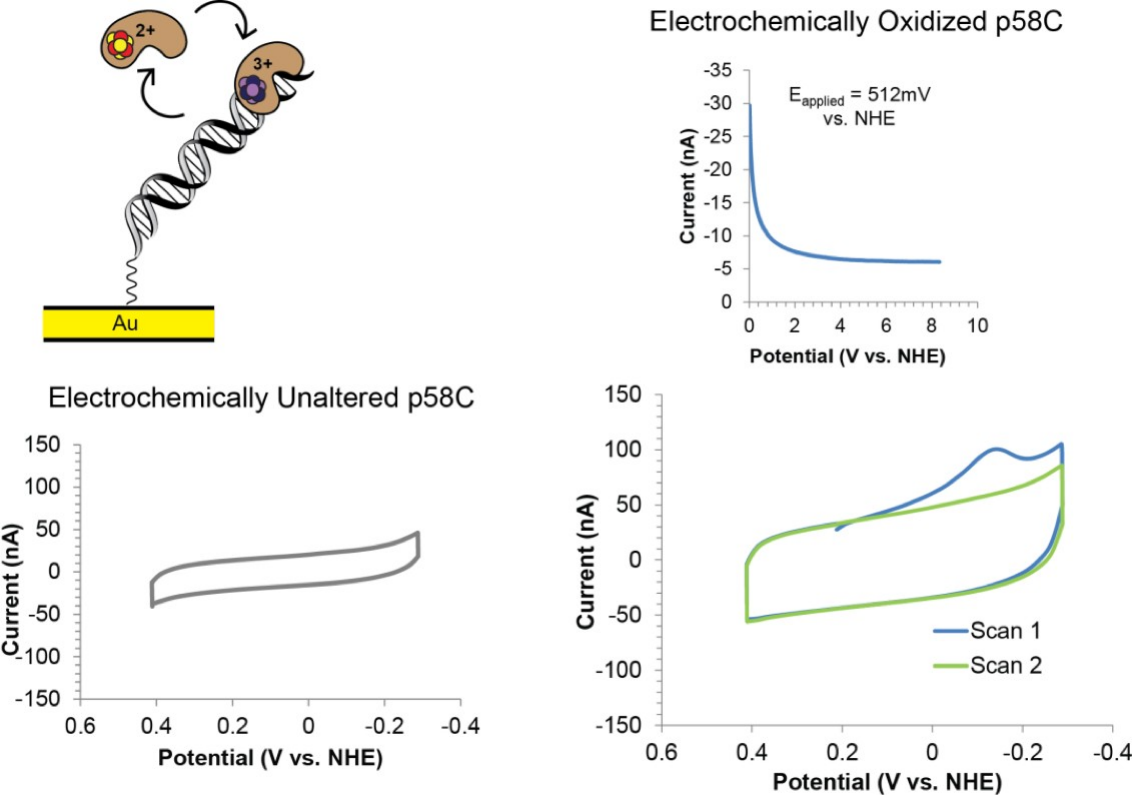
p58C_266-464_ participates in redox switching on DNA. The cartoon (top left) depicts p58C DNA binding and redox switching on an Au electrode. (Bottom left) CV scan of electrochemically unaltered p58C_266-464_. (Right) bulk oxidation (above) of p58C_266-464_ and subsequent CV scans (below). This construct displays similar electrochemical behavior to p58C_272-464_. All electrochemistry was performed in anaerobic conditions with 40 μM [4Fe4S] p58C_266-464_ in 20 mM HEPES (pH 7.2), 75 mM NaCl. CV was performed at 100 mV/s scan rate.

The [4Fe4S] cluster in p58C_272-464_ was previously shown to function as a redox switch regulating DNA binding and signaling activity [19]. The oxidized, [4Fe4S]^3+^ form of this protein was DNA-bound and redox-active; the reduced [4F-e4S]^2+^ form was loosely associated with DNA and exhibited no DNA-mediated electrochemical signal in a cyclic voltammetry scan. To test whether the same redox switching function was present in p58C_266-464_, we characterized this construct using DNA electrochemistry. Using the multiplexed, DNA-modified Au electrode platform shown in S3 Fig, we assessed the redox behavior of p58C_266-464_ on a 20-nt duplex DNA substrate with a 3-nt, 5’-ssDNA overhang [19]. Electrochemical experiments were performed in anaerobic conditions to prevent nonspecific degradation of the cluster by atmospheric oxygen [37]. Electrochemically unaltered p58C_266-464_, which is predominantly present in the [4Fe4S]^2+^ oxidation state, displays no redox signal on DNA, as observed with p58C_272-464_. However, upon electrochemical oxidation to the [4Fe4S]^3+^ state by bulk electrolysis at an applied potential of 512 mV vs. NHE, CV scans of p58C_266-464_ display a large reductive peak near -140 mV vs. NHE. As observed for p58C_272-464_, this peak disappears after one scan to negative reducing potentials (Fig 7). We next tested if the [4Fe4S] cluster of p58C_266-464_ functions as a redox switch and found that this construct can cycle between the tightly bound, redox-active [4Fe4S]^3+^ state, and the loosely associated [4Fe4S]^2+^ state. The p58C_266-464_ construct also displays a robust, reversible, NTP-dependent signal (S4 Fig) centered at 142 ± 12 mV vs. NHE on a DNA electrode. This is very similar to the signal observed for p58C_272-464_ in the presence of DNA and NTPs, the two necessary substrates for primase activity. Together, these data suggest that the two human p58C constructs, p58C_272-464_ and p58C_266-464_ have the same redox switching ability; the electrochemical function of the [4Fe4S] cofactor is consistent in both proteins.

## Discussion

It has been proposed that differences in the structures of residues Leu318-His351 in the crystal structures of p58C_266-456_ and p58C_272-464_ are due to the difference in their N-termini [20, 22]. It has been hypothesized that Ile271 provides critical hydrophobic interactions stabilizing the helical hairpin in the p58C_266-456_ crystal structure and that the absence of this residue leads to misfolding of Leu318-His351 in p58C_272-464_ and formation of β-strands in the p58C_272-464_ crystal structure. As these differences in structure are in the DNA-binding region of p58C, it is plausible to suggest that this may have significant functional repercussions. However, by determining the crystal structure of a new p58C_266-464_ construct with the same extended N-terminal sequence as the p58C_266-456_ construct, we demonstrated that the differences in the previously reported structures were not due to the difference in sequence, but rather to differences in the crystallization conditions.

Analysis of the p58C_266-464_ and p58C_272-464_ crystal structures reveals that substantial crystal lattice packing interactions stabilize this region in both structures. Specifically, Leu318-Gln329 are seen to interact extensively with the same region in the adjacent molecule in the lattice. The backbone atoms between the adjacent molecules in these regions show extensive hydrogen bonding, stabilizing the beta-like structure. In addition, on both sides of this extended interaction, Phe326 completes a hydrophobic pocket in the adjacent molecule, providing an additional anchor between the two molecules. In the structure of the p58C_266-456_ crystallized under different conditions, a series of stabilizing interactions involve Leu318-His351, but none of these are formed between adjacent molecules in the crystal lattice. A number of crystal contacts in this lattice are found in loop regions and the interfaces of hydrophobic helices. The differences between the crystal contacts in these two models can be attributed to differences in crystallization conditions, most notably the very high concentration of p58C (50 mg/mL) used originally for p58C_272-464_ [23] and now for p58C_266-464_. It is conceivable that at high concentration p58C forms dimers over time promoting the difference in crystal lattice and this in turn may facilitate formation of the inter-molecular Cys449 disulfide.

Crystallization contacts stabilizing alternate conformations of proteins is a well-demonstrated phenomenon [38]. Detailed comparative analysis of crystal structures, structural data obtained in solution, and simulations, have revealed a number of cases in which packing interactions affect backbone and side chain conformations, and even cases of alternate folds [39–41]. The addition of p58C to this group provides additional support for the importance of considering crystallization contacts between adjacent molecules in the crystal lattice when analyzing conformational changes of proteins crystallized with co-factors and ligands.

To further establish the equivalence of the p58C constructs, we turned to structural characterization in solution. Both CD and NMR (Figs 4 and 5) showed that the absence or presence of residues Gly266-Ile271 has no significant impact on structure in solution. DNA binding and cyclic voltammetry assays (Figs 6 and 7) showed that p58C_272-464_ and p58C_266-464_ have effectively the same biochemical properties. The evidence that the DNA binding region of p58C is capable of adapting to different crystallization conditions is consistent with the need for p58C to bind substrates sequence non-specifically, a property often associated with structural plasticity in sequence non-specific DNA binding domains (e.g. [42]). This raises the question: does the structure of p58C change upon DNA binding? One structure of p58C with a DNA substrate bound is available [12]. Comparison of this and the substrate-free structure reveals that only subtle changes in a limited number of side chains is required to bind the substrate [12, 14]. Given the extensive similarities between the p58C structures crystallized in different conditions, there is no reason to think the alternate conformation of residues 318–351 precludes DNA binding. Moreover, at a more fundamental level, it is the structure *in solution* with and without substrate that is required to definitely answer this question. Hence, further solution-state structural characterization of primase in complex with NTPs and primed substrates will be required to establish if there is a functional role for structural plasticity in the template-binding region of p58C.

We note that the approximately 3 μM dissociation constants we observe are similar to those previously reported using fluorescence anisotropy [19], but significantly higher than those reported for p58C_266-456_ binding to a similar substrate measured using an electrophoretic mobility shift assay (EMSA) [36]. Molecular oxygen is known to be generated while running a polyacrylamide gel, which may cause redox-active proteins to become oxidized over the course of an EMSA [32]. As p58C is proposed to bind substrates more tightly when oxidized, we suggest that the EMSA measurements may report the affinity of primarily oxidized p58C, whereas the fluorescence-based measurements report the affinity of the directly purified p58C, which is primarily reduced [9, 19]. Regardless of the differences in the K_D_ values measured in solution and with EMSAs, our data show the DNA binding affinities of p58C_272-464_ and p58C_266-464_ are effectively the same, indicating that the shorter construct functions normally.

All substrate binding measurements have been performed on p58C in the reduced state. Although a direct assessment of the substrate binding affinity of oxidized p58C is highly desirable, such experiments are precluded by the instability of the oxidized cluster. Use of chemical oxidants causes degradation of the cluster and precipitation of the protein, as does extended exposure to atmospheric oxygen. Moreover, although the oxidized protein can be generated electrochemically, the lifetime of the oxidized state is not sufficiently long to make a reliable direct measurement of DNA binding affinity by traditional methods. There is substantial interest in developing new approaches that would overcome this challenge.

In summary, we have shown that the absence or presence of residues Gly266-Ile271 does not lead to any significant differences in the structure and biochemical properties of the p58C domain of human DNA primase. Beyond addressing the concerns raised about our initial studies, these data strengthen the support for our proposal that the [4Fe4S] cluster serves as a redox switch that modulates the DNA binding affinity of p58C, setting the stage to further asses the role this switch plays in the priming function of human DNA primase.

## Supporting information

S1 Fig Domain map showing p58C constructs. (TIF)

S2 Fig The beta sheet-like region of p58C_266-464_ (red) is stabilized by interactions with the beta sheet-like region of an adjacent symmetry-related molecule (grey). (TIF)

S3 Fig Multiplexed, DNA-modified Au electrode platform used for p58C electrochemistry. Sixteen Au electrodes (circles, center) are divided into four quadrants, allowing for electrochemical measurements on different DNA substrates with replicates on a single surface.
(TIF)

S4 Fig p58_256-464_ Electrochemistry with 2.5 mM ATP. Upon addition of 2.5 mM ATP, a reversible redox signal generally appears in CV scans. The signal in the presence of 2.5 mM ATP was centered at an average midpoint potential measured near 142 ± 12 mV vs. NHE. The signal was observed at physiologically relevant redox potential, with a magnitude on the order of 10^2^ nC charge transport. All scans were performed under anaerobic conditions on 40 μM [4Fe4S] p58C in 20 mM HEPES (pH 7.2), 75mM NaCl, at 100mV/s scan rate (CV) or 15 Hz (SQWV).
(TIF)

S5 Fig Overlays of p58C_272-464_ and p58C_266-464_^15^N-^1^H HSQC NMR spectra. Overlay of the spectra of p58C_272-464_ (blue) and p58C_266-464_ (red) plotted in the same order (right) and in reverse order (left) relative to Fig 5. These spectra were acquired at 25°C on a Bruker AV-III spectrometer operating at 800 MHz. The samples contained 200 μM protein in a buffer containing 20 mM MES (pH 6.5), 50 mM NaCl, 2 mM DTT, and 5% ^2^H_2_O.
(TIF)

S6 Fig The p58C_266-464_ and p58C_272-464_ constructs do not bind fluorescein. **Titrations of** p58C_272-464_ (blue circles) and p58C_266-464_ (red squares) into 25 nM fluorescein in a buffer containing 20 mM MES (pH 6.5), 50 mM NaCl, 2 mM DTT, and 0.016% DMSO. Fluorescence anisotropy was measured at 25°C. Before plotting, the data were normalized by subtracting the fluorescence anisotropy value at [p58C] = 0 μM from each titration point. Error bars represent the standard deviation of three independent measurements.
(TIF)

S1 Dataset Circular dichroism data for p58C_266-464_ and p58C_272-464_. (XLSX)

S2 Dataset Processed HSQC data for ^15^N-labeled p58C_266-464_ and p58C_272-464_. (ZIP)

S3 Dataset Fluorescence intensity data for p58C_266-464_ and p58C_272-464_. (XLSX)

S4 Dataset Fluorescence anisotropy data for p58C_266-464_ and p58C_272-464_ and fluorescein control. (XLSX)

S5 Dataset Cyclic voltammetry data for p58C_266-464_ in the presence of 2.5 mM ATP. (ZIP)

S6 Dataset Bulk electrolysis and cyclic voltammetry data for oxidized p58C_266-464_. (ZIP)

S7 Dataset Cyclic voltammetry data for unaltered p58C_266-464_. (ZIP)
