# Supplementary figures and images for: Functional and structural similarity of human DNA primase [4Fe4S] cluster domain constructs
Source: PLoS One. 2018 Dec 18;13(12):e0209345. doi: 10.1371/journal.pone.0209345 (PMC6298731; doi:10.1371/journal.pone.0209345)

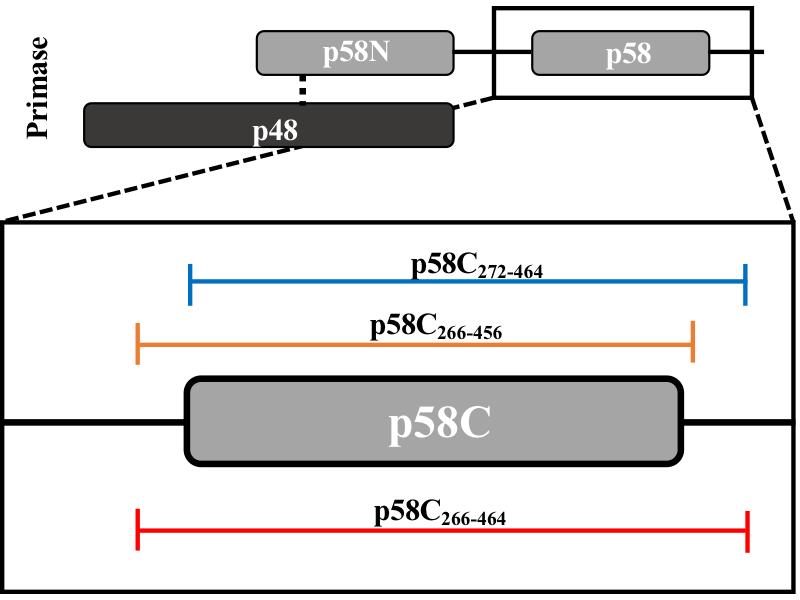

Supplement: S1 Fig — (TIF) [file pone.0209345.s001.tif]

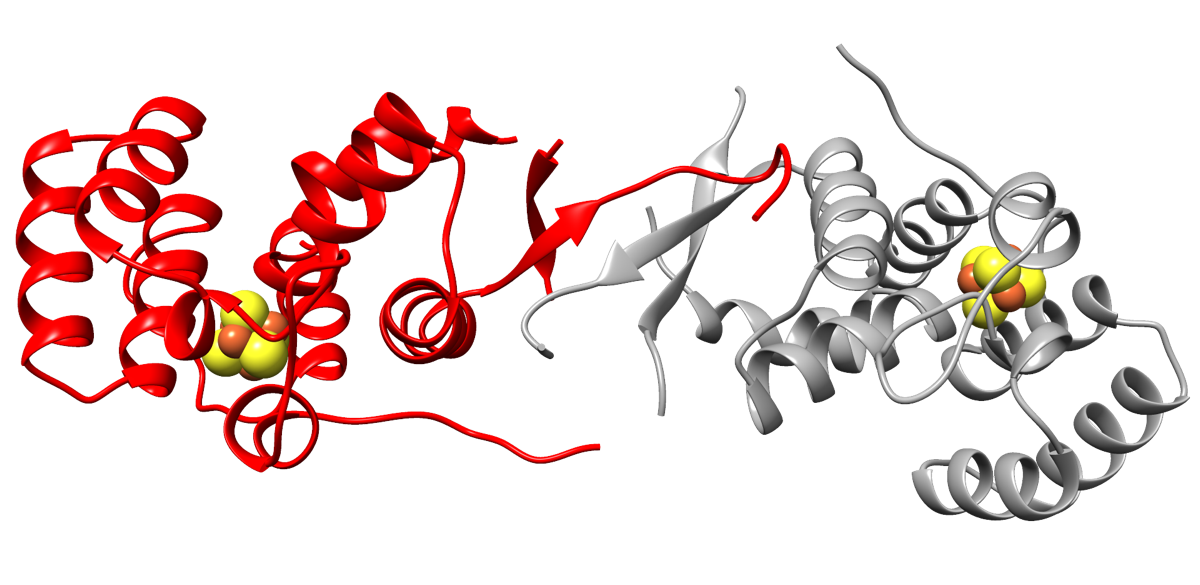

Supplement: S2 Fig — (TIF) [file pone.0209345.s002.tif]

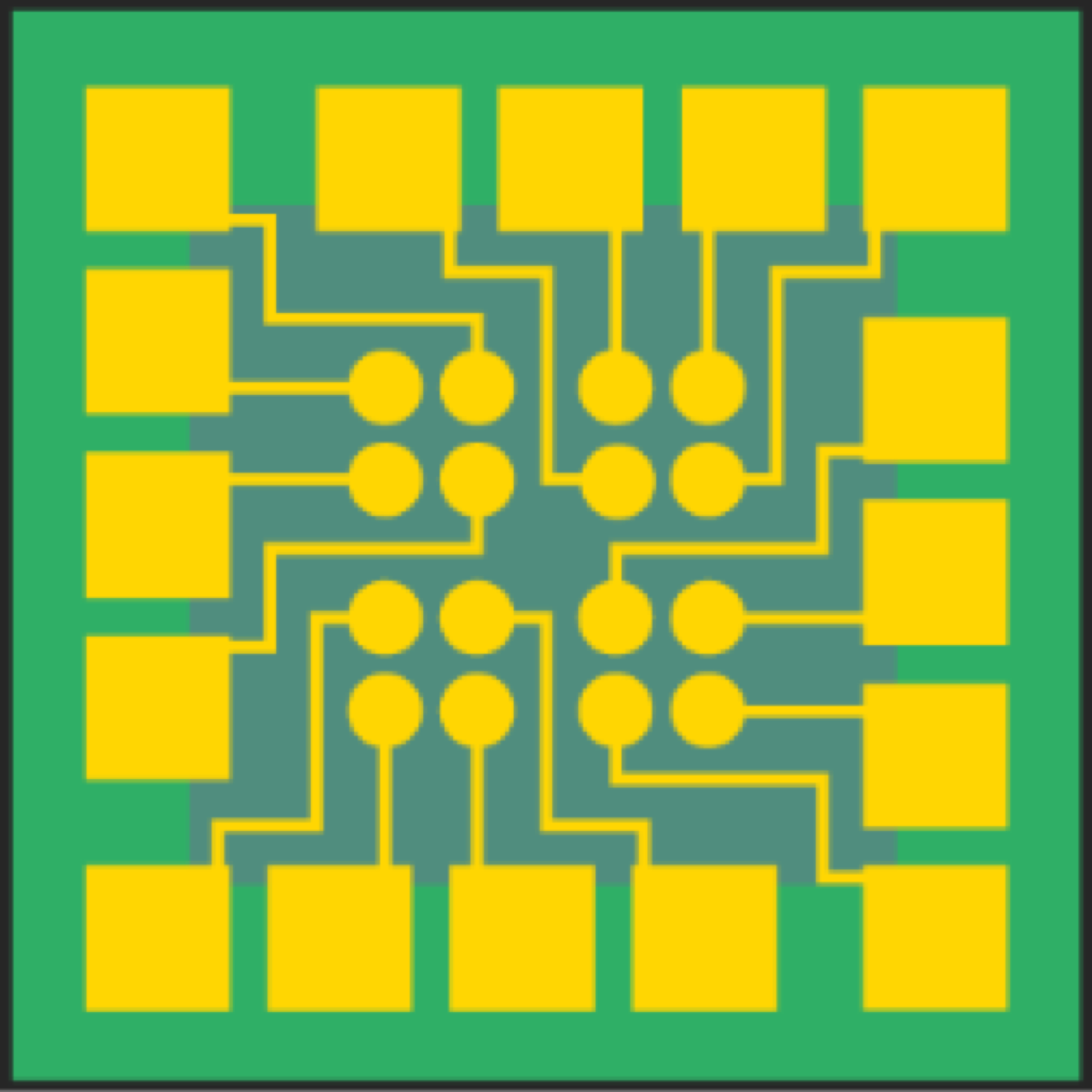

Supplement: S3 Fig — Sixteen Au electrodes (circles, center) are divided into four quadrants, allowing for electrochemical measurements on different DNA substrates with replicates on a single surface. (TIF) [file pone.0209345.s003.tif]

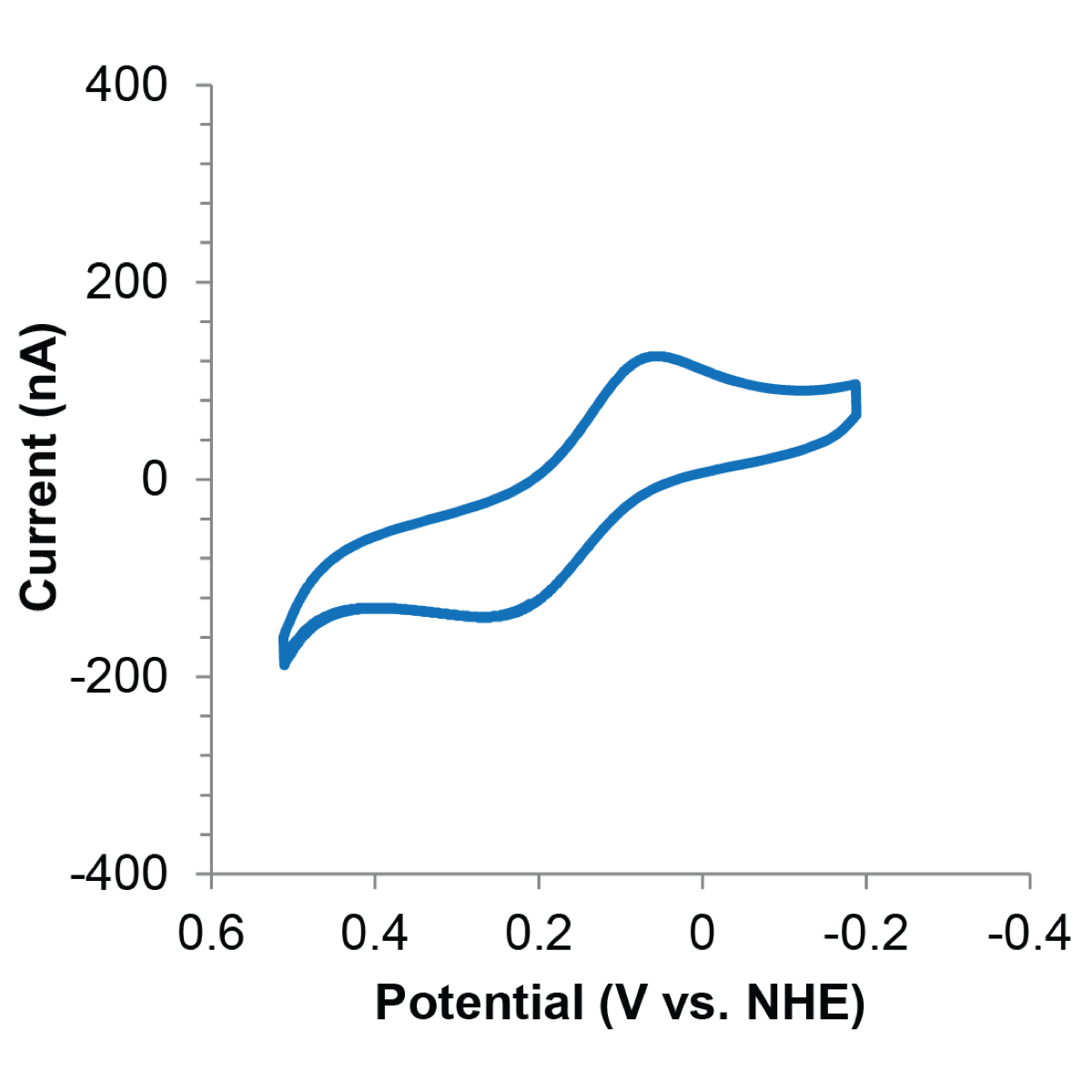

Supplement: S4 Fig — Upon addition of 2.5 mM ATP, a reversible redox signal generally appears in CV scans. The signal in the presence of 2.5 mM ATP was centered at an average midpoint potential measured near 142 ± 12 mV vs. NHE. The signal was observed at physiologically relevant redox potential, with a magnitude on the order of 102 nC charge transport. All scans were performed under anaerobic conditions on 40 μM [4Fe4S] p58C in 20 mM HEPES (pH 7.2), 75mM NaCl, at 100mV/s scan rate (CV) or 15 Hz (SQWV). (TIF) [file pone.0209345.s004.tif]

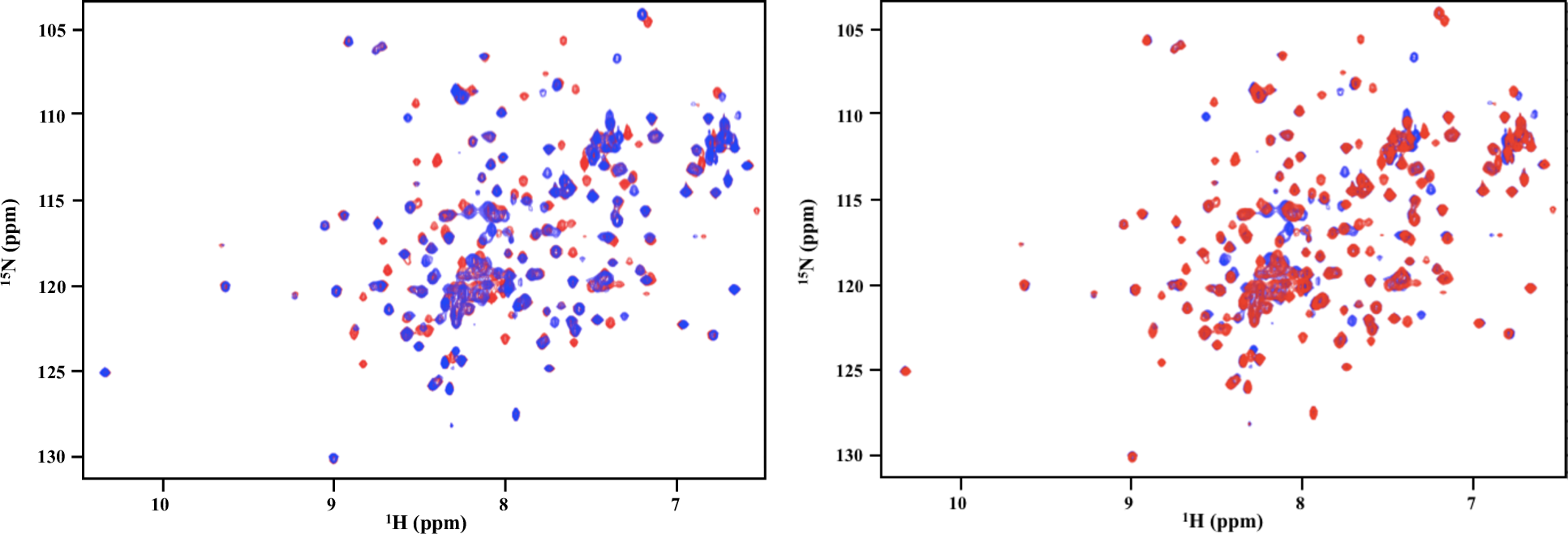

Supplement: S5 Fig — Overlay of the spectra of p58C272-464 (blue) and p58C266-464 (red) plotted in the same order (right) and in reverse order (left) relative to Fig 5. These spectra were acquired at 25°C on a Bruker AV-III spectrometer operating at 800 MHz. The samples contained 200 μM protein in a buffer containing 20 mM MES (pH 6.5), 50 mM NaCl, 2 mM DTT, and 5% 2H2O. (TIF) [file pone.0209345.s005.tif]

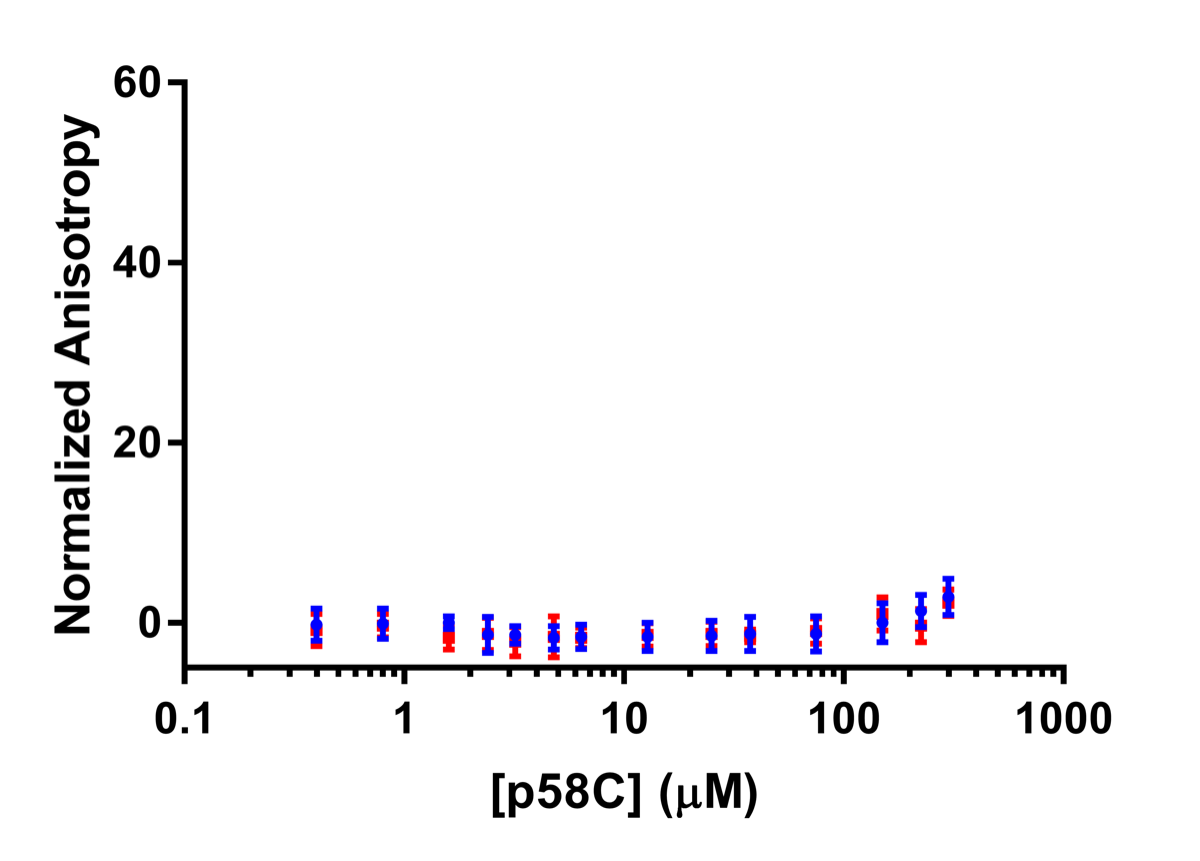

Supplement: S6 Fig — Titrations of p58C272-464 (blue circles) and p58C266-464 (red squares) into 25 nM fluorescein in a buffer containing 20 mM MES (pH 6.5), 50 mM NaCl, 2 mM DTT, and 0.016% DMSO. Fluorescence anisotropy was measured at 25°C. Before plotting, the data were normalized by subtracting the fluorescence anisotropy value at [p58C] = 0 μM from each titration point. Error bars represent the standard deviation of three independent measurements. (TIF) [file pone.0209345.s006.tif]
